# Development of a Microwave-Assisted Bench Reactor for Biomass Pyrolysis Using Hybrid Heating

**DOI:** 10.1021/acsomega.4c02050

**Published:** 2024-05-25

**Authors:** João C. S. Leite, Maria J. Suota, Luiz P. Ramos, Marcelo K. Lenzi, Luiz F. L. Luz

**Affiliations:** Department of Chemical Engineering, Federal University of Paraná, P.O. Box 19011, 81531-980 Curitiba, Paraná, Brazil

## Abstract

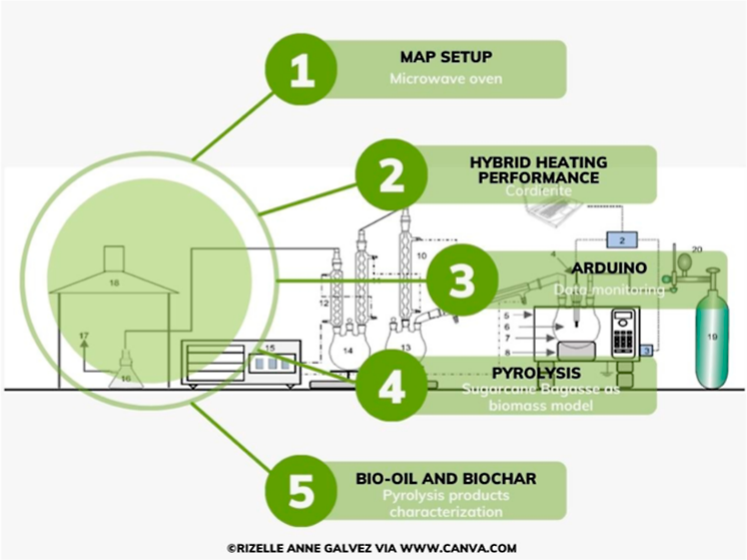

Microwave-assisted pyrolysis (MAP) is a cutting-edge technology that converts biomass into fuels, chemicals, and materials. In this study, an Arduino was used to control and automate a MAP system built from a microwave oven with a cordierite chamber filled with silicon carbide. Sugar cane bagasse was pyrolyzed at 250, 350, 450, and 550 °C to validate the MAP system and obtain pyrolytic products with different yields and chemical compositions. Lower temperatures led to high biochar yields, but the highest surface area of 25.14 m^2^ g^–1^ was only achieved at 550 °C. By contrast, higher temperatures favored the recovery of pyrolysis liquids. BET and scanning electron microscopy analyses revealed a porous biochar structure, while Fourier transform infrared spectroscopy analysis showed that the availability of functional groups on the biochar surface decreased with an increase in pyrolysis temperature. GC–MS analysis quantified valuable low molecular mass compounds in pyrolysis liquids, including aldehydes, ketones, phenols, and alcohols. With its unprecedented hybrid heating device, the MAP system promoted suitable heating rates (31.9 °C min^–1^) and precise temperature control (only 19 °C of set point variation), generating pyrolysis products devoid of microwave susceptor interferences. Therefore, MAP provided a rapid, safe, and efficient means of depolymerizing biomass, thus holding promise for biorefinery applications.

## 1 Introduction

Plant cell walls are a powerful renewable carbon source to produce fuels, chemicals, and materials that can potentially replace several fossil derivatives. Lignocellulosic materials are bulk renewable resources rich in biopolymers, such as cellulose, hemicelluloses, and lignin.^1^ These biopolymers are found in agricultural, agro-industrial, and forestry biomasses, such as barks, straws, cobs, stalks, shells, hulls, bagasse, and wood chips.

Sugar cane bagasse (SCB) is readily available in both sugar and ethanol industrial production units as a coproduct. Brazil produced 572.9 million tons of sugar cane in a harvested area of 8.34 million hectares in 2021/2022, generating around 174 million tons of residues.^2^ Each ton of sugar cane produces an average of 140 kg of SCB and 140 kg of agricultural sugar cane residues, such as straws and dry and green leaves, on a dry basis.^3^ In sugar and/or ethanol mills, most SCB is burned for heat, power, and electricity generation to help the energy balance of the industrial site. Instead, SCB can be used as a source of cellulosic derivatives, composites, biofuels, and high-value-added chemicals.^4^ A recent study suggested that SCB pyrolysis could contribute significantly to achieving several United Nations Sustainable Development Goals (SDGs), such as promoting sustainable agriculture and producing clean and accessible energy (SDG #2 and #7, respectively).^5^

Lignocellulosic materials can be depolymerized to produce platform chemicals such as carbohydrates, alcohols, ketones, phenols, aldehydes, and organic acids.^1^ Thermal routes have developed enormously in this field, providing a range of efficient pathways for biomass depolymerization. The main thermal conversion strategies include liquefaction, torrefaction, gasification, and pyrolysis.^6^ Among these, pyrolysis has excellent potential to improve the profitability, diversification, and sustainability of biorefineries based on primary residual resources such as SCB.

Pyrolysis processes produce biochar and pyrolytic liquids from biomass under an inert atmosphere.^7^ Numerous pyrolysis systems are disclosed in the literature with specific setups for different types of biomass.^8^ The heating source of a pyrolysis reactor can be either conventional or microwave-assisted (MAP). The latter has been recognized for its high-temperature set points and high heating rates, promoting relatively small temperature gradients and homogeneous heating.^9^ Several biomasses have been depolymerized using MAP, such as microalgae,^10^ lignin,^11^ and SCB.^12,13^

Yerrayya et al.^14^ used a multimode on–off controlled 480 W microwave oven for lignin pyrolysis in various microwave carbonaceous susceptors. Selective depolymerization in high yields was achieved for phenolic compounds using activated carbon (AC) as the absorber using lignin to susceptor mass ratio of 1:9. Also, Aziz et al.^15^ performed the pyrolysis of agricultural wastes at 250–390 °C in short reaction times (2–7 min) using a 1000 W domestic microwave oven to produce bio-oils. Therefore, MAP systems present several possibilities for design, construction, and temperature measurement that can be set based on the properties of the raw material to be pyrolyzed and the desired target temperatures.

Lignocellulosic materials are often weak microwave absorbers. This explains how challenging the promotion of high heating rates in MAP systems is. Indeed, lignocellulose pyrolysis requires absorbent materials to improve heat transfer, such as AC and silicon carbide (SiC), also known as thermal catalysts.^14,16^ However, their usage is somewhat complicated because they are often mixed with biomass to be pyrolyzed.^17^ Hence, absorber separation becomes difficult after pyrolysis and may result in low purity in biochar recovery. Besides, absorbers are responsible for hotspots that can damage the reactor walls and thermocouples, and eventually cause uncertainties in temperature measurements. Ex situ absorbers seem more advantageous because the pyrolytic solids remain free from the absorber at the end of the pyrolysis procedure. Additionally, further steps are not necessary for biochar collection and separation.

In this study, a MAP unit was developed using an unprecedented hybrid heating system to provide high heating rates without any absorber-biomass contact. SCB, a well-known and abundant agroindustrial residue in tropical countries, was used as a reference material for testing the MAP system performance.

## 2 Materials and Methods

### 2.1 Materials

SCB was kindly provided by Raízen (Piracicaba, SP, Brazil) with the following chemical composition: 3.7 ± 0.5% ash, 5.6 ± 0.4% extractable materials, 35.8 ± 0.8% glucans, 21.3 ± 0.7 xylans, 1.5 ± 0.2 arabinose residues, 4.3 ± 0.1 acetyl groups, 25 ± 2 total lignin, and 13.2 ± 1.5% moisture. The SCB ultimate analysis revealed 46.7% carbon, 6.0% hydrogen, 0.28% nitrogen, sulfur slightly above the detection limit, and 47.0% oxygen by difference using an Elementar Vario MICRO Cube analyzer (Langenselbold, Germany).^18^

SCB was ground in a bench-scale knife mill (Willey mill) to particle sizes within 28–48 Mesh before pyrolysis. SiC (98.85%, 40 Mesh—Saint-Gobain, France), a heat absorber, and a thermal insulant cordierite block were employed to assemble the heating system. N_2_ (99.98%) was purchased from White Martins (Brazil) to provide an inert atmosphere and flow the volatiles. Dichloromethane (DCM) (99.8%—Merck, Brazil) was used as the washing solvent for collecting the liquid phase. Anhydrous sodium sulfate (99%—Synth, Brazil) was used as a drying agent before chemical and chromatographic analysis.

### 2.2 Experimental Setup

**Figure 1:**
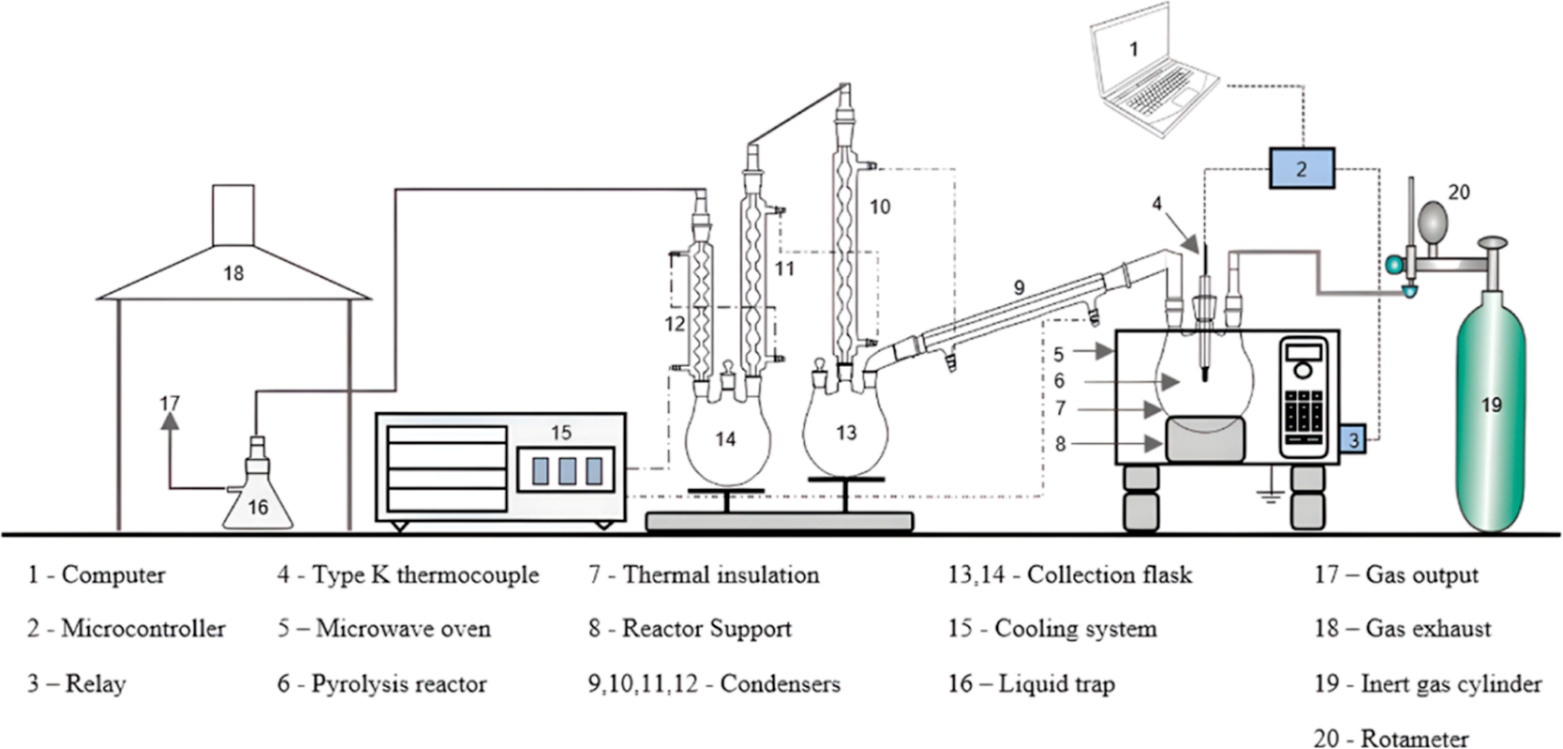
Schematic illustration of the MAP components.

The schematic diagram of the MAP system is shown in Figure 1. The bench reactor was custom-built using a 900 W, 2.45 GHz Panasonic microwave oven (Kadoma, Japan), model NN-S52BH (28 L), with the inverter technology. The microwave oven was drilled following the safety protocol presented by Mushtaq, Mat, and Ani.^19^

**Figure 2:**
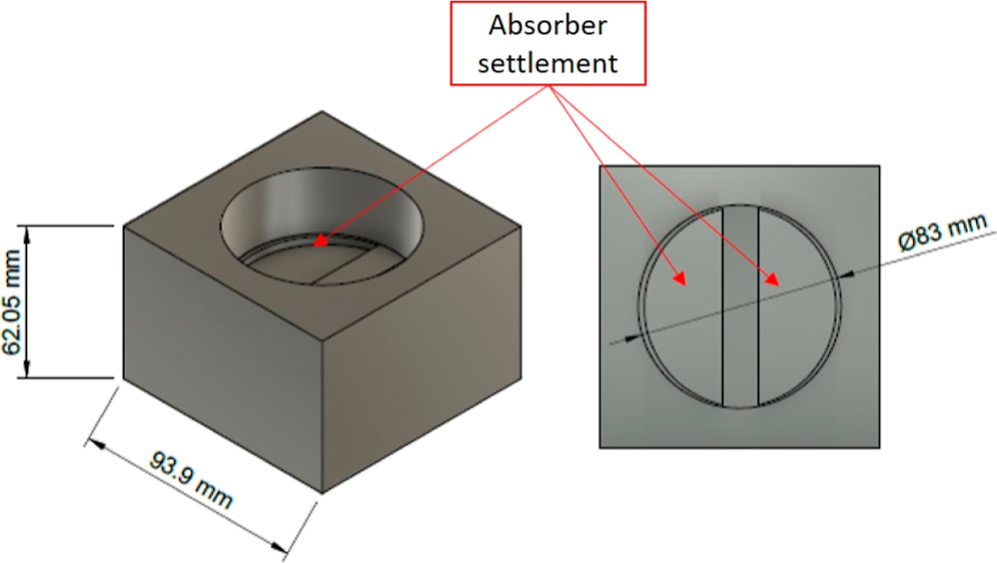
Hybrid-heating appliance for heat generation upon microwave irradiation.

A hybrid heating apparatus was built for the reactor support using a thermally insulating cordierite brick and 98.85% SiC (40 Mesh) as the microwave absorber. SiC was placed carefully at the bottom of the support parallel to the waveguide output (Figure 2). In addition, the reactor was wrapped with glass wool to improve system insulation. Packing the absorber (SiC) at the bottom of the cordierite brick reduced the occurrence of hot spots and improved heating homogeneity (Figure S2). In addition, this hybrid heating system allowed the sample to be irradiated simultaneously by the microwave energy and the convective heat emanating from SiC. No deformation or cracking of the reactor walls was observed because the glassware was not in direct contact with the superheated surface of SiC. A K-type thermocouple was placed inside the reactor for in situ temperature measurements and heating control using a converter module (MAX6675) with a temperature range of ±2.25 °C. Also, the system counted on a model SSR25 (25 A, 24 to 380 VAC) solid-state relay (Fotek), a 0.96 in. I2C OLED display (Organic Light-Emitting Diode), and a Mega 2560 Arduino microcontroller.

Generally, laboratory-scale MAP systems use quartz reactors due to their low thermal expansion and structural stability at temperatures up to 1000 °C.^15,20^ However, quartz reactors are costly. As an alternative, this work employed borosilicate glass flasks as reactors due to their lower cost and great commercial availability. In addition, borosilicate glass flasks can be used for pyrolysis when the reaction temperature is below 600 °C. A 500 mL borosilicate glass round-bottom flask (used as the reactor vessel) was connected to a 500 mm long straight condenser and a series of three 600 mm long ball condensers cooled to 2 °C by a thermostatic bath (Tecnal model TE-184). The condensed liquids were collected in two 500 mL three-necked round-bottom flasks. The oxygen-free environment was provided by coupling a N_2_ gas cylinder to one of the three openings of the reactor vessel (Figure 1). The MAP assembly, including the insulation device and other accessories, is shown in Figure S1 of Supporting Information. The reactor was fixed at the top of the microwave reactor using clamps, providing an easy-to-assemble arrangement and insulation to avoid thermal losses.

The C/C++ programming language was used to develop the controlling system, and the data was stored in a MEGA 2560 microcontroller by Arduino IDE (Integrated Development Environment). This microcontroller could trigger and interrupt the relay, handle the thermal data collected by the thermocouple, send the data as readable information to the OLED display, and register the pyrolysis data in an Excel spreadsheet through a PLX-DAQ interface (Parallax Inc.).

The On/Off temperature control used in this work is very convenient since it requires simple software and inexpensive hardware. However, temperature measurements were not accurate and difficult to control due to thermal inertia. This led to rising temperatures even after the magnetron was automatically switched off. Thus, a “Look-Ahead Temperature” (LAT) mathematical model was developed to predict the temperature increase and overcome this bottleneck. The LAT considered the measured temperature and multiplied it by a predetermined temperature factor expressed by eq 1
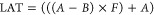
1where *A* is the measured temperature, *B* is the previous temperature, and *F* is the multiplication factor. The resulting value is then compared to the preconfigured reaction temperature (set point), and the magnetron is switched off when the system exceeds the specified temperature. Once the set point was reached, the magnetron reclosing was no longer linked to the predicted value (T look ahead) but to the actual system temperature, following a recurrent logic. The glass wool reactor insulation avoided losing thermal energy, helping keep the set point temperature. According to Wang et al.,^17^ power measurements were carried out by adding 500 g of distilled water into the reactor chamber and heating it under 20, 40, 70, and 100% of the nominal microwave power (NMP) to 100 ± 2 °C.

### 2.3 Factorial Design and Pyrolysis Conditions

A 2^4^ factorial design was carried out in triplicate for process preoptimization, in which the temperature, the microwave power, the amount of absorber, and the N_2_ gas flow were varied (Table 1). Initially, the MAP system was purged with N_2_ for 5 min before 30 g were irradiated with 100% NMP until the set temperature (250, 350, or 450 °C) was reached. Afterward, the power was reduced to 20% NMP, and pyrolysis ran up to 23 min when the system was shut down and cooled to room temperature under N_2_ flow.

**Table 1:** Experimental Set for the MAP of SCB

experiment code	temperature (°C)	NMP^a^ (%)	absorber (g)	N_2_ flow (L/h)
450–100–60–3	450	100	60	3
450–40–60–3	450	40	60	3
450–40–60–9	450	40	60	9
450–100–60–9	450	100	60	9
250–40–60–9	250	40	60	9
350–70–30–6-A^b^	350	70	30	6
250–100–60–3	250	100	60	3
250–40–60–3	250	40	60	6
350–70–30–6-B^b^	350	70	30	6
350–70–30–6-C^b^	350	70	30	6
250–100–60–9	250	100	60	3
450–100–0–3	450	100	0	3
450–40–0–9	450	40	0	9
450–100–0–9	450	100	0	9
450–40–0–3	450	40	0	3
250–40–0–3	250	40	0	3
250–100–0–3	250	100	0	3
250–100–0–9	250	100	0	9
250–40–0–9	250	40	0	9

a Nominal microwave power.

b Central point triplicate.

The biochar was collected and weighed. The pyrolysis liquids were removed from the MAP system using DCM as the washing solvent. Then, DCM was removed under reduced pressure to constant mass. The gaseous phase was calculated by difference and neglected for analysis. The glass reactor was heated at 380 °C in a muffle furnace for cleaning and to avoid possible cracking in the subsequent experiments. After understanding the influence of process variables based on the proposed experimental design (Table 1), a new set of experiments was carried out in triplicate using a 30 g sample and the following pyrolysis conditions: 100% NMP (644.70 W), 60 g SiC, and 3 L h^–1^ N_2_ at the programmed temperatures of 250, 350, 450, and 550 °C.

### 2.4 Effect of Pyrolysis Temperature on Bio-oil Yield

Understanding the influence of pyrolysis parameters on product yields plays a crucial role in improving process efficiency. After the experimental screening proposed in Table 1, the conditions for the best bio-oil yield were reproduced in triplicate at 250, 350, 450, and 550 °C. The collection of bio-oil and biochar followed the same procedure depicted in Section 2.3.

### 2.5 Fourier Transform Infrared Spectroscopy Analyses

Biochar was analyzed using Fourier transform infrared (FTIR) spectroscopy. The spectra were obtained in a Bruker Vertex spectrophotometer (Billerica, USA) with 32 scans at a resolution of 2 cm^–1^ in the 4000–400 cm^–1^ wavenumber range. Biochar samples were dispersed in KBr (0.7 wt %), and disks were prepared for FTIR analysis in the transmission mode.

### 2.6 Specific Surface Area Measurement

Specific surface areas were determined by N_2_ adsorption/desorption at 77 K after sample degasification for 3 h at 200 °C. Measurements were carried out in a Quantachrome NOVA 1200 instrument (Boynton Beach, USA) using the Brunauer–Emmett–Teller (BET) method.

### 2.7 Morphology by Scanning Electron Microscopy

Scanning electron microscopy (SEM) micrographs were used to characterize the morphology of the biochar particles. Biochar samples were metalized by Au/Pd sputtering and analyzed with an acceleration potential of 20 kV and magnification of 500× for imaging acquisition. The equipment employed was a microscope JEOL JSM-6010LA (Tokyo, Japan) available at the Mineralogy Laboratory (LAMIR) of UFPR. SEM images were acquired using the proprietary JEOL software.

### 2.8 GC–MS Analysis

Bio-oils (heavy bio-oils) were extracted from crude pyrolysis liquids using DCM. The remaining aqueous solution containing the polar phase (light bio-oil) was obtained by solid-phase extraction (HyperSep C18 Cartridges), followed by DCM elution (see Supporting Information for additional details). Both samples were derivatized using *N*,*O*-bis(trimethylsilyl)trifluoroacetamide (BSTFA) and analyzed by GC–MS using a Shimadzu QP-2010 chromatograph (Kyoto, Japan) with a VF-5MS capillary column (30 m × 0.25 mm; 0.25 μm). Each sample (1 μL) was injected using a 1:10 split ratio and run at 50 °C for 2 min. Then, the temperature was increased from 5 °C min^–1^ to 280 °C, where it remained for 2 min. The ion source was kept at 200 °C and the interface at 280 °C. The eluted compounds were identified by comparing their mass spectra with those of the NIST11 Mass Spectral Library. Similarity indexes below 80% were disregarded. A semiquantitative analysis was performed by area normalization for compounds with an abundance higher than 1%. Primary standards were used for qualitative and quantitative analysis when available.

## 3 Results and Discussion

### 3.1 Monitoring and Control of the MAP System

The MAP system was controlled and monitored through an Arduino EXCEL computer interface and an IDE spreadsheet (see Figure S3 in Supporting Information). Both set point and absolute pyrolysis temperatures were monitored through an Arduino luminous display protected from electromagnetic interference by a metal box and grounded for safety. Also, the electromagnetic field interference on temperature measurements was avoided by isolating the thermocouple using a glass sheath placed into a stainless-steel shield (Figure S4).

**Figure 3:**
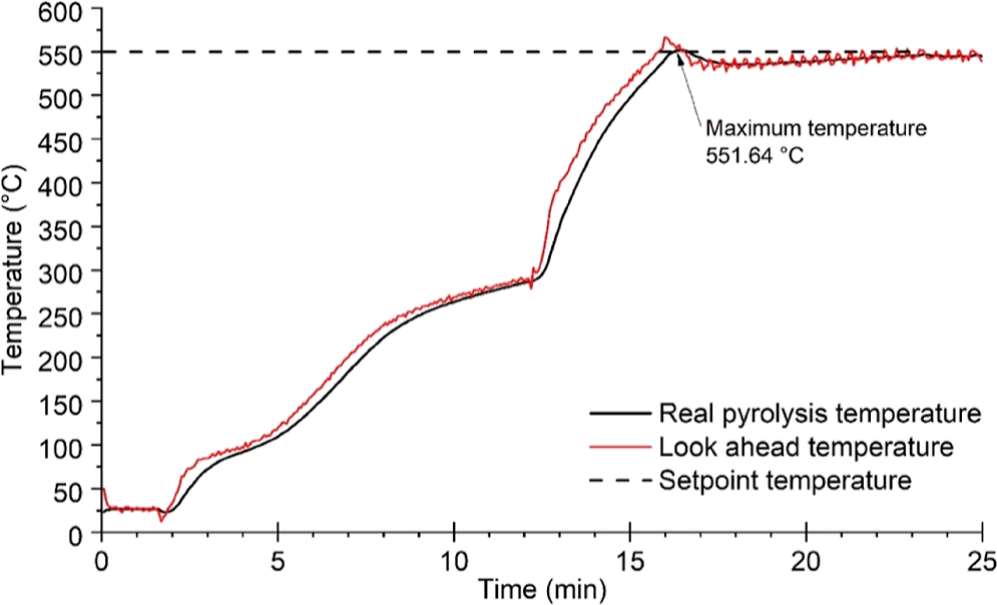
Layout of the MAP monitoring system with the set point temperature, the real pyrolysis temperature, and the predicted temperature.

Figure 3 depicts the LAT predictive function for a pyrolysis experiment with a set point of 550 °C. The maximum and minimum temperatures reached after the process shutdown were 551.6 and 534.8 °C, respectively. LAT demonstrated itself as a suitable tool for forecasting and keeping the desired temperature since the heating system was turned off automatically after reaching the predicted temperature.

The NMP of 950 W was tested in triplicate at 20, 40, 70, and 100% levels to measure the heating system’s efficiency. The observed power was 227.9 ± 3.2, 440.0 ± 2.3, 562.2 ± 3.4, and 644.7 ± 2.9 W, respectively. These findings align with Wang et al.,^17^ in which the observed power was 48–80% of the actual NMP value.

### 3.2 Analysis of Process Variables

The results from the 2^4^ factorial design expressed the influence of temperature, power, absorber amount, and N_2_ flow on the yield of pyrolysis products (Table 2).

**Table 2:** Temperature, Heating Rate, and Product Yields for MAP of SCB

experiment code	temperature (°C)	heating rate (°C min^–1^)	biochar^a^ (%)	bio-oil^b^ (%)	pyrolysis gas (%)
450–100–60–3	464.7	31.9	24.0	50.6	25.3
450–40–60–3	376.7	16.2	30.6	48.6	20.6
450–40–60–9	464.5	22.7	26.3	46.6	27.0
450–100–60–9	456.4	25.1	24.3	46.0	29.6
250–40–60–9	262.9	13.9	38.0	42.0	20.0
350–70–30–6	313.8	13.4	38.6	41.0	20.3
250–100–60–3	271.2	34.7	46.3	40.0	13.6
250–40–60–3	254.3	15.7	40.0	38.3	21.6
350–70–30–6	287.6	12.3	51.3	34.6	14.0
350–70–30–6	282.5	12.1	46.3	34.3	19.3
250–100–60–9	266.8	20.0	42.3	33.3	24.3
450–100–0–3	277.1	11.9	60.6	27.3	12.0
450–40–0–9	218.0	9.5	63.3	25.3	11.3
450–100–0–9	280.7	12.0	64.0	25.3	10.6
450–40–0–3	249.9	10.7	62.3	24.0	13.6
250–40–0–3	243.1	10.4	63.3	23.6	13.0
250–100–0–3	252.2	21.5	69.6	19.3	11.0
250–100–0–9	249.6	11.6	73.6	17.0	9.3
250–40–0–9	230.2	9.8	75.3	15.3	9.3

a Referred as the solid of the pyrolysis process.

b Bio-oil included the light and heavy bio-oils.

The highest biochar yield (75.3%) was achieved without SiC using 230.2 °C, 40% NMP, 9 L h^–1^ N_2_ flow, and a heating rate of 9.8 °C min^–1^ (experiment 250–40–0–9). Only 15.3% of pyrolysis liquids (bio-oil) were obtained under these experimental conditions, showing that biochar yield increases in inverse proportion to bio-oil. By contrast, the highest bio-oil yield (50.67%) was obtained at 464.7 °C using 644.70 W, 60 g SiC, 3 L h^–1^ N_2_ flow, and a heating rate of 31.9 °C min^–1^ (experiment 450–100–60–3), a condition in which 100% NMP and a microwave absorber were employed. In addition, the use of high heating rates contributed to the production of more pyrolysis liquids. Since both reaction temperature and heating rates are influenced by microwave power and the use of SiC as a microwave absorber (Table 2), modulation of these two parameters could effectively promote electric energy savings.

The use of microwave absorbers is critical for MAP. Except for experiment 250–100–0–3, which had a low set point temperature, higher-set point temperature experiments in the absence of a microwave absorber did not reach the temperature. For instance, in experiment 450–40–60–3, the SCB was irradiated with 40% NMP using 60 g of SiC, and these conditions enabled it to reach only 376.7 °C.

### 3.3 Effect of Absorber Amount on Temperature Profiles

**Figure 4:**
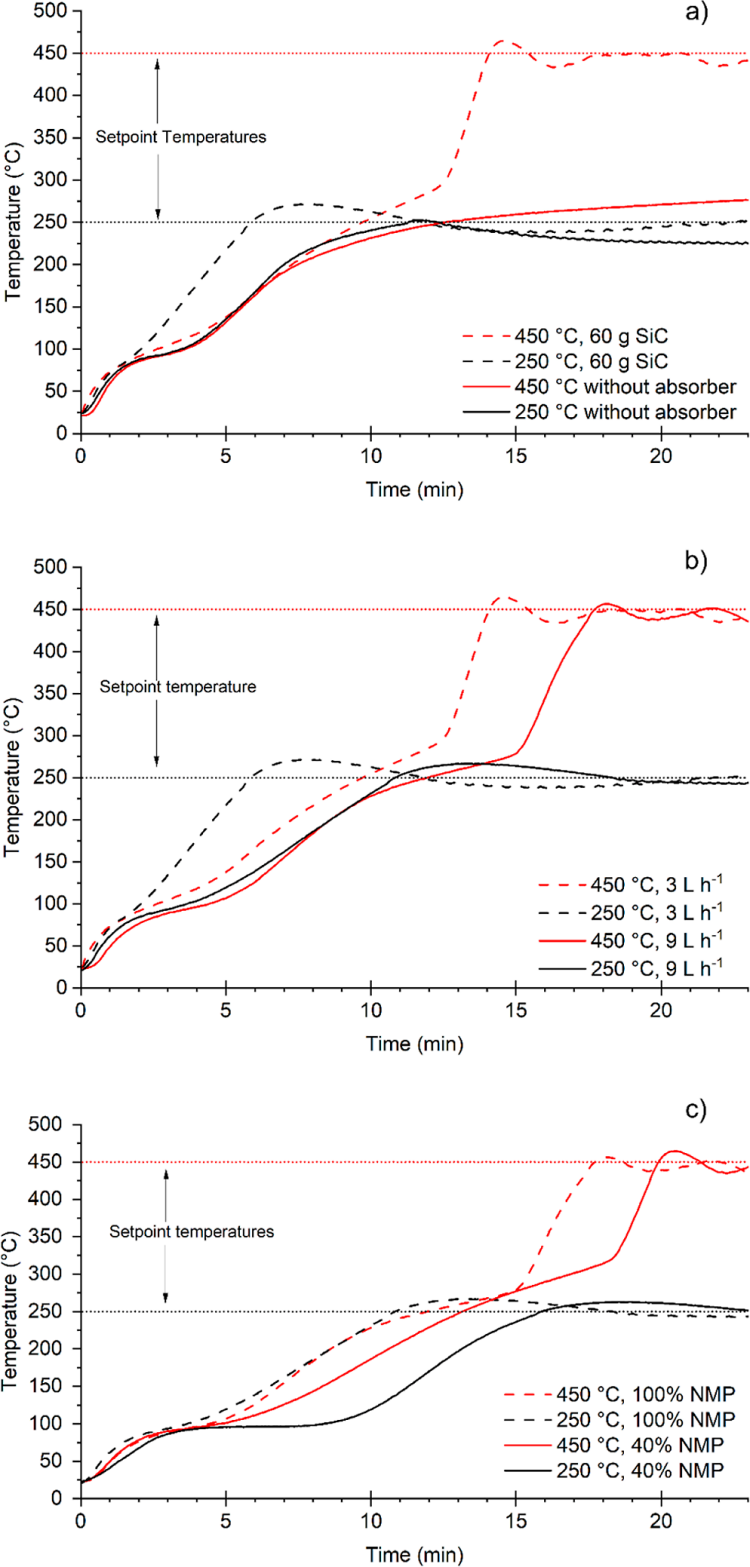
(a) Effect of microwave absorber (SiC), (b) N_2_ flow, and (c) microwave power on the heating profile using 250 and 450 °C as the target temperature.

Figure 4a shows the MAP heating profiles for experiments performed at 250 and 450 °C using 100% NMP and 3 L min^–1^ N_2_ flow.

Absorbers modify temperature profiles and increase heating rates in MAP systems.^21^ The absence of SiC showed inefficient heating and prevented pyrolysis from reaching the set point temperature. By contrast, with 60 g of SiC, the system continued to heat up to the third pyrolysis stage, which was well above 250 °C. In this case, energy transfer continued from the heating source (absorber) and by microwave energy absorption. Zhao et al.^22^ suggested a fast mass loss at the third pyrolysis stage and evidenced a similar heating profile for the MAP of corn stalks.

The heating rate and bio-oil yield in experiment 450–100–60–3 were 31.9 °C min^–1^ and 50.6%, respectively. In the absence of the absorber, these values were 11.9 °C min^–1^ and 27.33%, respectively. Thus, the absorber was crucial for producing a reasonable heating rate, which was critical for producing a high bio-oil yield. The effects of the type and quantity of the absorber are not well explored in the MAP of SCB, compared to other factors such as feedstock loading and particle size, moisture content, catalyst dosage, microwave power, and reaction time.^5,12^ Toscano Miranda et al.^5^ reported that absorbers improve heat transfer from the reactor to the biomass particles by conduction, providing high heating rates that contribute to reaching the target temperature. Nevertheless, further studies are still required to demonstrate the viability of such MAP systems on a large scale.

### 3.4 Effect of N_2_ Flow on Temperature Profiles

Besides promoting a nonreactive atmosphere, the inert gas flow provided different residence times for gases and vapors inside the pyrolysis reactor. Also, the inert gas may have affected the temperature dynamics and product yields.^23^ At a low N_2_ flow, the biomass was rapidly heated up to the set point temperature (Figure 4b). For instance, 450 °C was reached in 14 min using N_2_ at 3 L h^–1^, while 17 min were needed to reach 450 °C at a 9 L h^–1^ N_2_ flow. The same behavior was observed for the set point temperature of 250 °C. The delay in reaching the set point temperature can be explained by the residence time of the gas inside the reactor chamber. Also, the injection of N_2_ at room temperature might have contributed to reducing the heating rate and extending the time needed to reach the desired temperature.

Bio-oil yields were higher at lower N_2_ flows. By contrast, the N_2_ flow had no apparent influence on biochar yields. By comparison, an expressive amount of the pyrolysis gas was released even at MAP at lower temperatures and higher N_2_ flow rates (Table 2).

Parihar et al.^24^ studied the relationship between N_2_ flow and temperature during the conventional pyrolysis of SCB at 450 °C using a fixed bed tubular reactor. Bio-oil yields of 46.2 and 51.1 wt % were obtained using N_2_ flows of 3 and 12 L h^–1^, respectively. In this study, higher N_2_ flows slightly decreased bio-oil yields, which may be attributed to differences in reactor design.

The heating was quicker at lower N_2_ flows. Thus, a high N_2_ flow must have cooled the pyrolysis reactor, causing some in situ vapor condensation and leading to a lower liquid recovery in the collection flask. Secondary cracking and polycondensation can occur in this condition, favoring pyrolysis gas and biochar yields.

### 3.5 Effect of Microwave Power on Temperature Profiles

The temperature profiles in Figure 4c reveal the influence of using low (40%) and high (100%) NMP for the pyrolysis of SCB at the same N_2_ flow and absorber amount (9 L h^–1^ and 60 g SiC, respectively). Both target temperatures of 250 and 450 °C were reached in shorter times using 100% NMP. However, the temperature profiles below 100 °C were almost the same upon any applied power. This is because biomass loses moisture up to 100 °C, intensifying the interaction between water and microwaves. Therefore, the applied power mainly influenced the temperature profiles after drying. Leite et al.^25^ found that the yield and quality of SCB pyrolysis products are affected by moisture content. Biochar produced at 13% moisture content has a higher porosity and yield than that produced at 3%.

Selvam and Paramasivan^12^ evaluated the influence of several process parameters in the MAP of SCB for biochar production, including moisture content. SCB batches with 8 and 16% moisture yielded 13.7 and 23.8% biochar at the same pyrolysis conditions, respectively. In addition, microwave power and reaction time significantly influenced the product yield. According to the authors, the surface temperature is maximum at higher microwave power, and the heating rate is faster, facilitating the pyrolysis reaction, especially due to the biomass’s small particle size. By contrast, no pyrolysis occurred at low microwave power (360 W), even after adding 30% carbon as an absorber. Therefore, the electric field was not intensive enough to reach the desired temperature under low power.

### 3.6 Pyrolysis Yields Using the Selected Reaction Conditions

**Figure 5:**
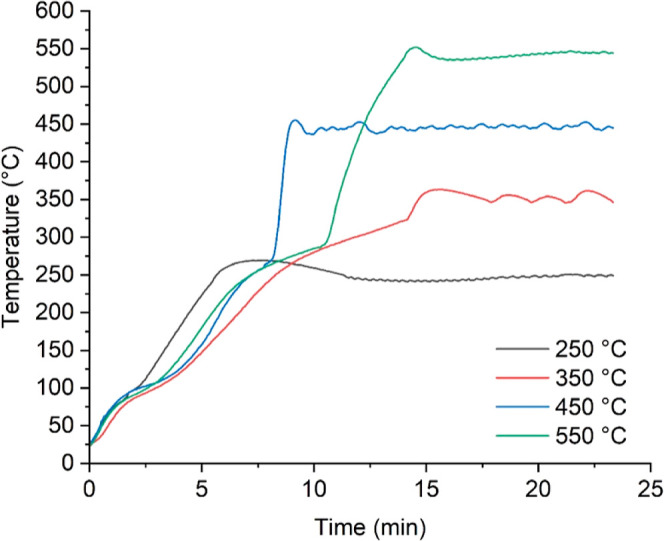
Temperature profiles for the MAP of SCB at 250, 350, 450, and 550 °C using 100% NMP, 60 g SiC, and 3 L h^–1^ N_2_ flow.

After understanding the influence of SiC, microwave power, and N_2_ flow on reaction performance, MAP was carried out under the following conditions: 100% NMP (644.70 W), 60 g SiC, and 3 L h^–1^ N_2_. Experiments were performed with 30 g in triplicate at the programmed temperatures of 250, 350, 450, and 550 °C, which resulted in the actual temperatures of 269.3 ± 3.7, 363.2 ± 11, 455.4 ± 1.5, and 551.6 ± 5.2 °C, respectively. Hence, the deviations beyond the set point were 19.3, 13.2, 5.3, and 1.6 °C, respectively, showing that temperature adjustment was more effortless above 450 °C (Figure 5). Contrastingly, experiment 350–50–60–3 was carried out at 60% NMP because 100% could comprise system stability and eventually exceed the set point temperature.

**Figure 6:**
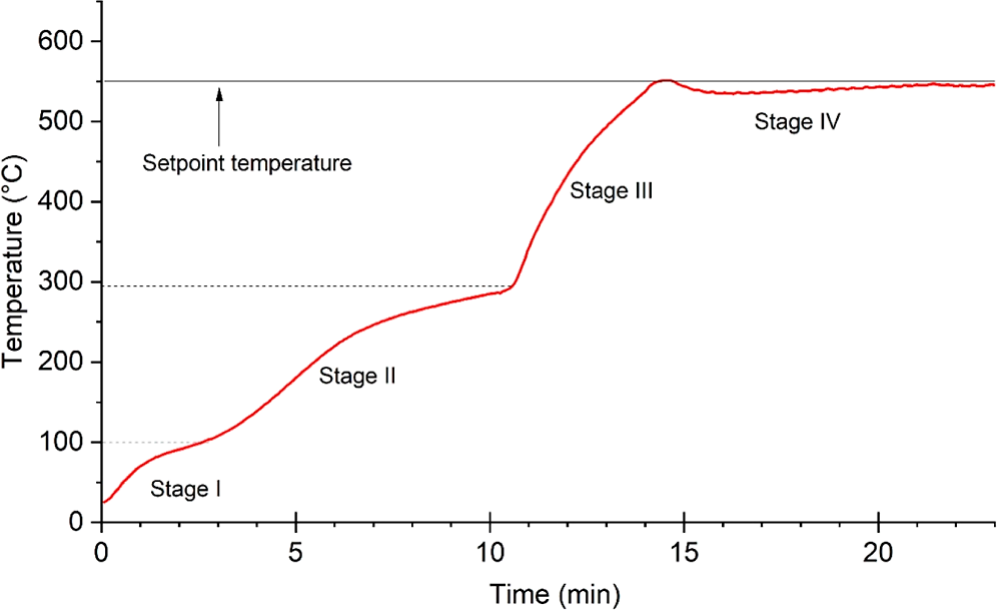
Temperature profile for SCB MAP using 100% NMP, 60 g SiC, and 3 L h^–1^ N_2_ flow.

The temperature control in MAP systems is sensitive to different pyrolysis reaction stages. Zhao et al.^22^ pyrolyzed corn stalk bales and obtained a MAP curve of four steps. The first step comprised water evaporation around 100 °C; between 180 and 200 °C, the prepyrolysis took place where the stalk bales were heated at a low heating rate; in the third step, the temperature rose quickly, promoting a relatively fast mass loss; and, finally, thermal equilibrium was reached at the set point temperature. By comparison, the prepyrolysis stage was less perceptible for the SCB since it was heated quickly due to the lower capacity of the reactor. Overall, the MAP developed in this study followed the same behavior (Figure 6), indicating coherence with other robust pyrolysis reactors already reported in the literature.

SCB loses water as an endothermic peak at temperatures below 150 °C. Then, hemicellulose decomposition occurs at 220–315 °C, followed by cellulose degradation ranging from 355 to 400 °C.^5^ Thermogravimetric analysis has shown two main thermal events at 315 and 353 °C, confirming these biopolymers’ thermal decomposition (Figure S5 in the Supporting Information). Hemicelluloses undergo depolymerization to produce 1,4-anhydro-d-xylopyranose and furfural, whereas cellulose forms d-glucopyranose (1,6-anhydroglucose) and levoglucosan. By comparison, lignin has the most complex and slowest decomposition process, attributed to its complex macromolecular structure. Its decomposition occurs in a vast range of temperatures and usually generates phenolic compounds.^26^ All these components come together to form the liquid pyrolysis products, along with biochar and noncondensable volatiles depending on the operation regime.

**Figure 7:**
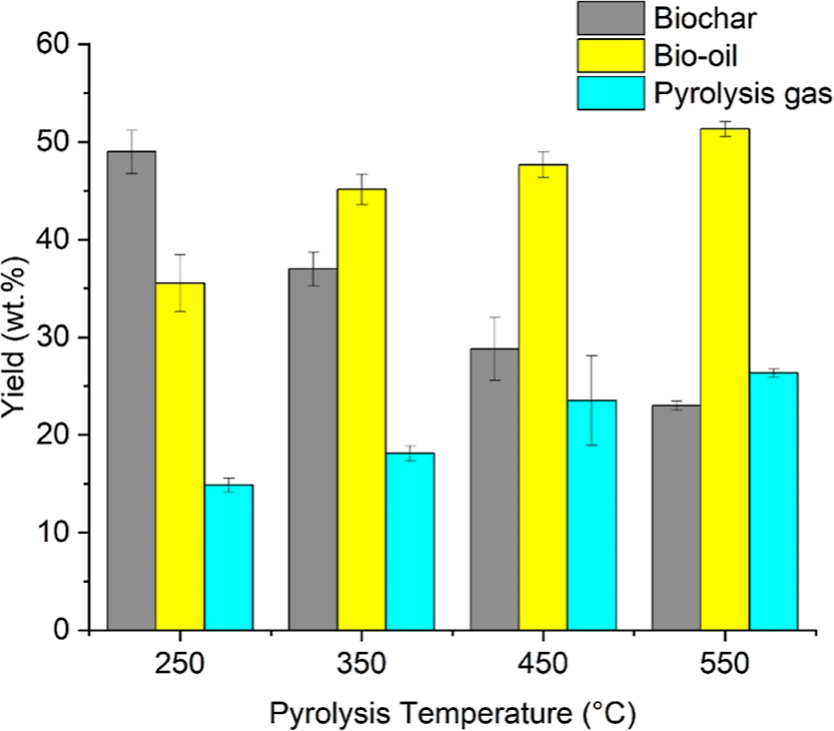
Yield of biochar, bio-oil, and pyrolysis gas produced at 250, 350, 450, and 550 °C using 100% NMP, 60 g SiC, and 3 L h^–1^ N_2_ flow.

Figure 7 depicts the biochar, bio-oil, and pyrolysis gas yields obtained by MAP. The biochar yield decreased toward higher pyrolysis temperatures, while bio-oil and pyrolysis gas followed the opposite trend.

The drop in biochar yield can be primarily explained by the primary decomposition of biomass at high temperatures and the secondary decomposition of intermediates formed during pyrolysis.^27^ The secondary decomposition of biochar at high temperatures can also explain the increasing yield of pyrolysis gas since secondary cracking usually generates noncondensable gases.^28^

### 3.7 Fourier Transform Infrared Spectroscopy

**Figure 8:**
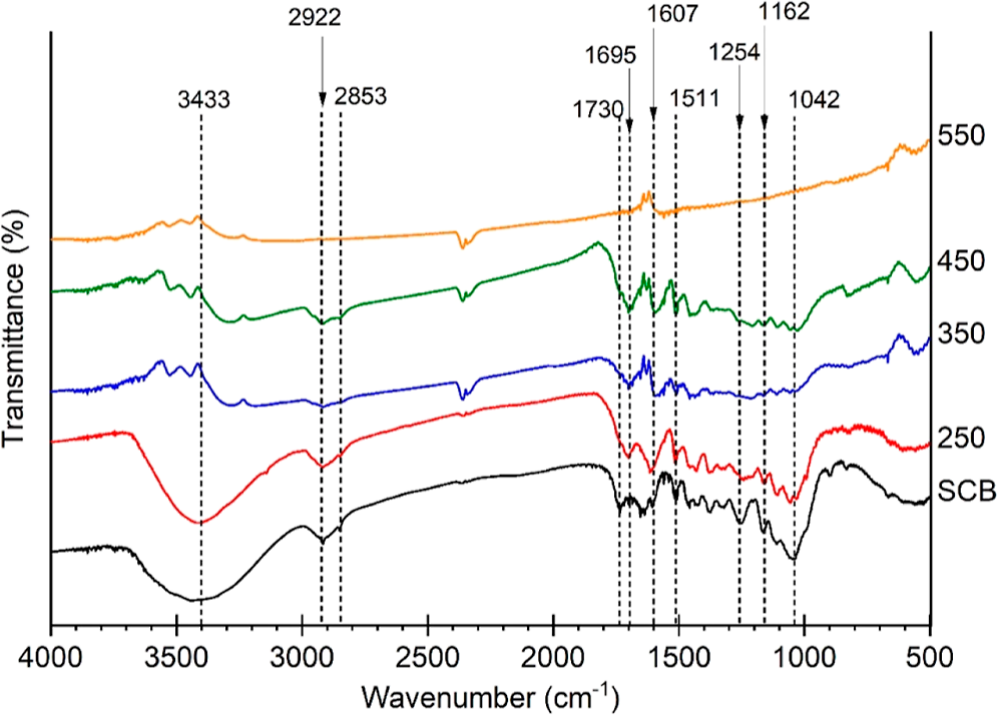
FTIR spectra of raw SCB and biochars produced under several pyrolysis temperatures using 100% NMP, 60 g SiC, and 3 L h^–1^ N_2_ flow.

FTIR analysis revealed the behavior of functional groups under thermal stress at different MAP temperatures. Compared to raw SCB, pyrolysis at high temperatures reduced the intensity of O–H axial deformations (around 3433 cm^–1^) in the biochar FTIR spectra (Figure 8). Such decrease is often attributed to dehydration and decarboxylation reactions that indicate a certain level of degradation in the polymeric structures of carbohydrates and lignin.^29^

The symmetrical and asymmetrical axial deformation of C–H in CH_2_ and CH_3_ are located at 2922 and 2853 cm^–1^, respectively. These bands gradually disappeared with an increase in pyrolysis temperature, suggesting degradation of both lignin side chains and carbohydrates.^30^ The bands at 1730 cm^–1^ refer to the C=O axial deformation of carboxylic acids, which probably indicates uronic acid in hemicelluloses.^31^ By contrast, the C=O axial deformation at 1695 cm^–1^ may be attributed to carbonylic compounds associated with unsaturated structures such as double bonds and aromatic rings. The bands at 1607, 1500, and 1430 cm^–1^ are typical of the skeletal vibrations of aromatic rings in lignin. However, the latter may also be partially attributed to C–H deformations.^29^ Typical C–O stretching bands at 1254 cm^–1^ refer to phenolic groups of lignin.^32^ In contrast, the prominent band in the SCB band at 1042 cm^–1^ may be assigned to the axial deformation of C–O in primary alcohols. The remaining bands are associated with the symmetric stretching of the glycosidic bond (−C–O–C−) in pyranose rings in carbohydrate chains. These bands decreased their intensity with gradual temperature increments from 250 to 550 °C, suggesting, once again, the partial degradation of lignin and cellulose (Figure 8). These observations are consistent with a previous study about peanut shell pyrolysis using in situ FTIR and thermogravimetric analysis coupled to FTIR and mass spectrometry (TG-FTIR-MS), in which biomass pyrolysis mechanisms were investigated at the molecular level.^29^

### 3.8 Specific Surface Area (BET)

N_2_ adsorption analysis revealed the textural properties of the biochars. Properties such as morphology, specific surface area, pore size distribution, average pore diameter, and pore volume qualify biochars for potential industrial uses, particularly for adsorption applications. MAP at 250 °C produced biochars with undetectable surface area (Table 3). By contrast, high pyrolysis temperatures increased specific surface area and enlarged both biochars’ average pore diameter and pore volume^33−35^

**Table 3:** Textural Characteristics and BET-Specific Surface Area of Biochars

biochar sample	B.E.T specific surface area (m^2^/g)	pore volume (cm^3^/g)	pore diameter average (Å)
250–100–60–3	nd^a^	nd^a^	nd^a^
350–100–60–3	4.90	3.314 × 10^–3^	2.70
450–100–60–3	3.11	3.438 × 10^–3^	2.21
550–100–60–3	25.14	1.972 × 10^–2^	3.14

a not detected.

Similarly, high heating rates are expected to induce meso and macropores formation.^36^ Biochar samples presented a well-structured mesoporous surface from the sudden volatile compound evolution. At 450 °C, the specific surface area of biochars (3.1 m^2^/g) was similar to that obtained from corn stalk (4.2 m^2^/g), pinewood (5.2 m^2^/g), and algae (3.3 m^2^/g) using a scaled-up MAP reactor at 400 °C.^37^ Surprisingly, 550 °C produced biochars with a specific surface area 8-fold higher than 450 °C. By comparison, corn stalk, pinewood, and algae biochars have not produced high specific surface areas such as this, even at 600 °C.^38^ Indeed, pyrolysis of SCB at a set point of 650 °C produced biochar with an even greater specific surface area and pore volume, three times higher than those obtained at 550 °C. However, its average pore diameter remained almost the same. This result is part of an independent experiment to assess how high pyrolysis temperature could influence the pore structure development (unshown data). Commercial AC has specific surface areas higher than 500 m^2^ g^–1^. Hence, an activation process could be used to achieve higher biochar-specific surface areas.

### 3.9 Scanning Electron Microscopy

**Figure 9:**
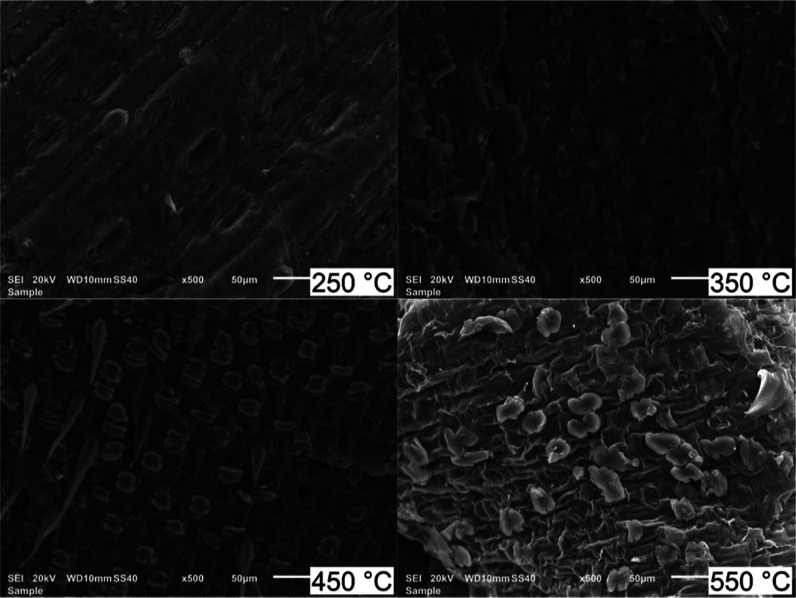
SEM micrographs (500×) of biochar produced at different pyrolysis temperatures (250, 350, 450, and 550 °C) using 100% NMP, 60 g SiC, and 3 L h^–1^ N_2_ flow.

The SEM helped characterize the morphology of SCB biochars produced at different pyrolysis temperatures (Figure 9). MAP at 250 °C preserved the structure of stomata and vessels of the biomass, evidencing the presence of organic matter. However, when the temperature was increased by 100 °C, erupted stomata were observed more often, indicating the release of volatile materials. Large pores were not generated in SCB biochar even at 550 °C. This finding is aligned with the textural analysis of these samples, which evidenced a structure predominantly mesoporous. Selvam and Paramasivan^12^ obtained an SCB porous biochar surface in the form of honeycomb and rough structures. By contrast, the biochars produced in this work had a rough surface with no evidence for a honeycomb formation. From a pore analysis viewpoint, biochars obtained at 550 °C and above could be suitable for adsorption applications and an important biomaterial for water retention.^33^

### 3.10 GC–MS Analysis

Pyrolysis of lignocellulosic materials generates hundreds of substances that condense as a water-rich viscous liquid called bio-oil. Besides an expressive amount of water, lignocellulosic bio-oils usually contain low-molar-mass alcohols, ketones, aldehydes, carboxylic acids, and phenolic compounds in their composition.^1^ While aliphatic ketones, aldehydes, alcohols, furfural, and furans are derived from cellulose and hemicelluloses,^39^ aromatic compounds such as phenols, cinnamic acids, aromatic ketones, and aldehydes arise primarily from lignin degradation.^40^

Ghorbannezhad et al.^41^ used sulfuric and formic acids, hot water, and microwave-assisted hot water to pretreat SCB before pyrolysis. Besides changing SCB chemical composition, such pretreatment techniques altered the bio-oil composition. Whereas acid pretreatments favored aromatic hydrocarbons and C_5_–C_12_ olefins production, hot water and microwave-assisted hot water (MW-HW) pretreatments generated C_9_–C_12_ alkanes and furans as major compounds. Thus, a tunable bio-oil composition is possible depending on the pretreatment employed in the conversion process. Similarly to our study, several common compounds, including catechol, pyrocatechol, levoglucosan, and vanillin, were found in the pretreated SCB pyrolysis oil. However, light and heavy bio-oils were not distinguished in this study, which is relevant for further separation and application of bio-oil.

**Figure 10:**
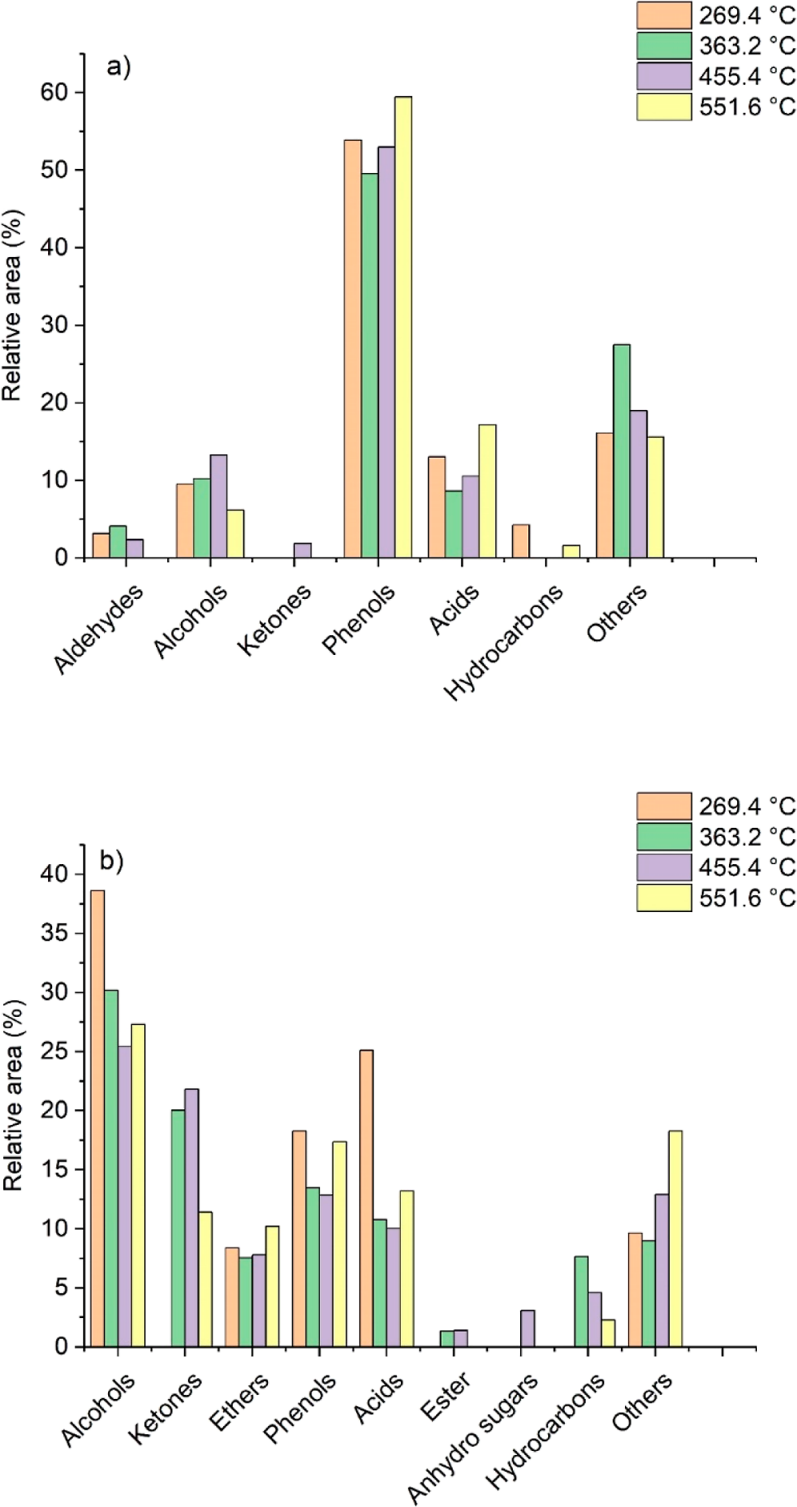
Composition of heavy bio-oils produced by SCB MAP at 250, 350, 450, and 550 °C after (a) liquid–liquid extraction (heavy bio-oils) and (b) solid-phase separation (light bio-oils).

GC–MS analysis revealed the complex chemical composition of SCB pyrolysis oils. The main components in heavy and light bio-oils were identified based on the similarity of their mass spectra with the NIST11 library. Afterward, these components were grouped into prominent organic families (alcohols, ketones, ethers, phenols, acids, esters, sugars, hydrocarbons, aldehydes, and others), as depicted in Figure 10.

Heavy bio-oils contain mainly low-molar-mass phenolic components, such as guaiacol, syringol, and eugenol. Also, 3-ethylphenol, 2-methoxyphenol, and 3-methoxyphenol have been identified as lignin degradation products (Figure 10a). This finding evidenced that the MAP system was very effective for biomass conversion, especially for lignin depolymerization, which possesses high thermal stability.^32^

The light bio-oil mainly contained alcohols, ketones, phenolic compounds, and organic acids (Figure 10b). Besides being acidic, light bio-oils were yellowish and less viscous than dark-brown heavy bio-oils due to their much higher water content. Water binds strongly to oxygenated polar compounds in bio-oil, which are hard to remove.^42^ Alcohols derive mainly from hydroxylated components of the lignocellulosic matrix. The presence of alcohols was higher in light bio-oils produced at lower temperatures (269.4 °C), but their concentration decreased with an increase in MAP temperature. Furfuryl alcohol resulting from carbohydrate thermal degradation was identified and confirmed by an authentic standard. By contrast, the lack of authentic standards for other pyrolysis products from biomass remains a bottleneck to be solved. In total, 72.3, 71.0, 67.7, and 86.4% of the light bio-oil compounds and 80.7, 72.5, 79.1, and 84.4% of the heavy bio-oil compounds produced at 250, 350, 450, and 550 °C, respectively, were identified with 80% of similarity with the NIST11 library. Compounds grouped as “others” could not be adequately identified due to their low similarity index. However, only minor compounds were included in this group. Naturally, the sample complexity and eventual coelution of peaks can make identifying and quantifying the bio-oil compounds difficult. Therefore, bidimensional chromatography techniques, such as GC–GC–MS, could be used to improve bio-oil characterization.

## 4 Conclusions

A pyrolysis unit that uses microwaves for heating was successfully developed and tested to break down SCB by noncatalytic pyrolysis at temperatures up to 550 °C. The heating support that combined a hybrid system with the Arduino platform was critical for an efficient and accurate temperature measurement. The system effectively converted SCB into biochar, bio-oil, and pyrolysis gas. Lower temperatures yielded more biochar, but high surface areas were only achieved at 550 °C. BET and SEM analyses revealed that biochars had a porous structure, while FTIR analysis showed that the availability of functional groups decreased at increased reaction temperatures. Also, higher temperatures led to a better recovery of pyrolysis liquids, reaching more than 50% at 550 °C. A vast range of valuable compounds was identified in pyrolysis liquids. Heavy bio-oils comprised 55% phenols, while light bio-oils presented more than 30% alcohols. Therefore, the MAP unit can support future research in biomass valorization, bioenergy, and biomaterials generation.

## Data Availability

The data will be made available upon reasonable request.

## Supporting Information Available

Illustrations of elements of the MAP system and TGA/DTG curves for SCB (PDF)
