# Supplementary material for: Development of a Microwave-Assisted Bench Reactor for Biomass Pyrolysis Using Hybrid Heating
Source: ACS Omega. 2024 May 25;9(23):24987–97. doi: 10.1021/acsomega.4c02050 (PMC11170622; doi:10.1021/acsomega.4c02050)

SUPPORTING INFORMATION

Development of a microwave-assisted bench reactor for biomass pyrolysis using hybrid heating

João C. Segatto Leite[1], Maria J. Suota[1], Luiz P. Ramos[1], Marcelo K. Lenzi[1], Luiz F. L. Luz Jr.[1]*

[1]Department of Chemical Engineering, Federal University of Paraná, P. O. Box 19011, Curitiba, PR, 81531-980, Brazil

CONTENTS

1. Elements of the MAP system
2. Thermogravimetric Analysis of sugarcane bagasse

*maria.juliane@ufpr.br, Orcid: 0000-0002-7879-2373

Figure S1. a) Assembly, b) set up, and c) insulation of the pyrolysis reactor

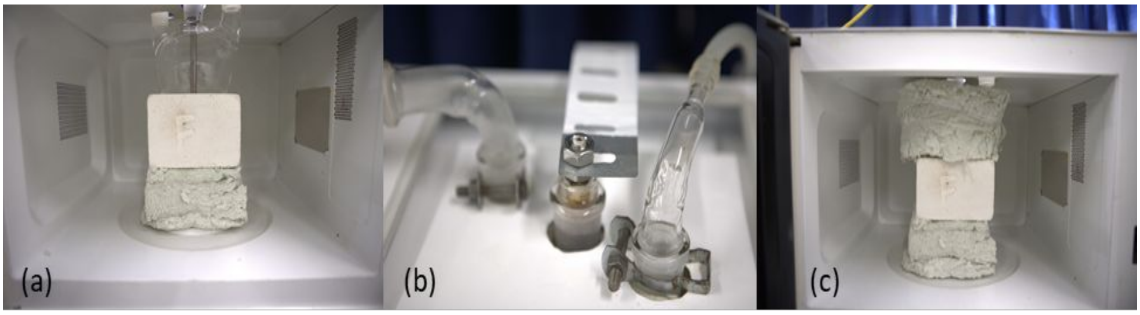

Figure S2. Cordierite support with SiC safely arranged for efficient heating

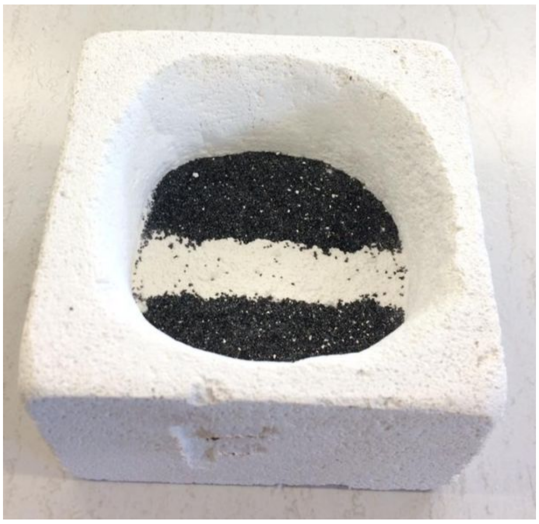

Figure S3. Primary elements of MAP control and monitoring: a) computer interface, b) Arduino top view, and c) Arduino luminous display

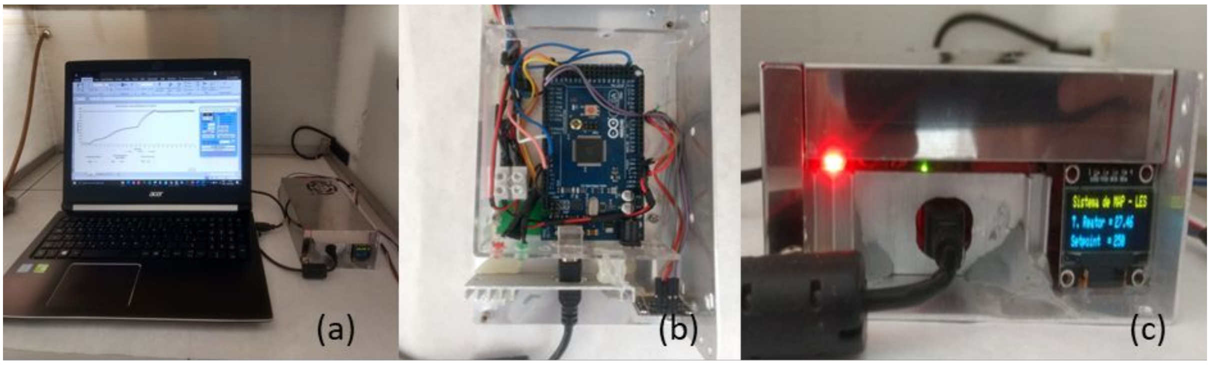

Figure S4. Thermocouple and its metallic protection

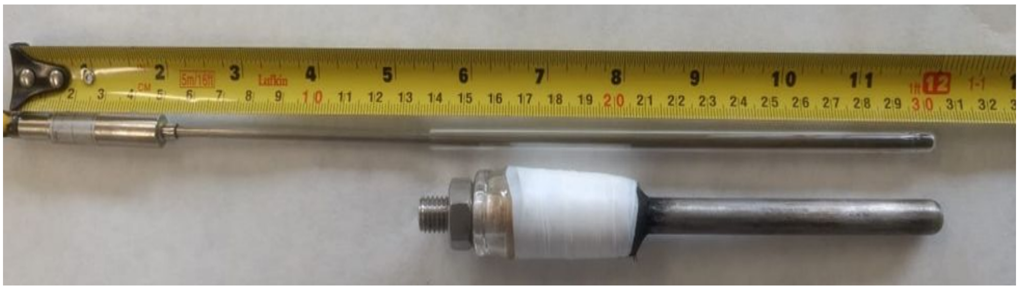

Figure S5. TGA/DTG curves for sugarcane bagasse

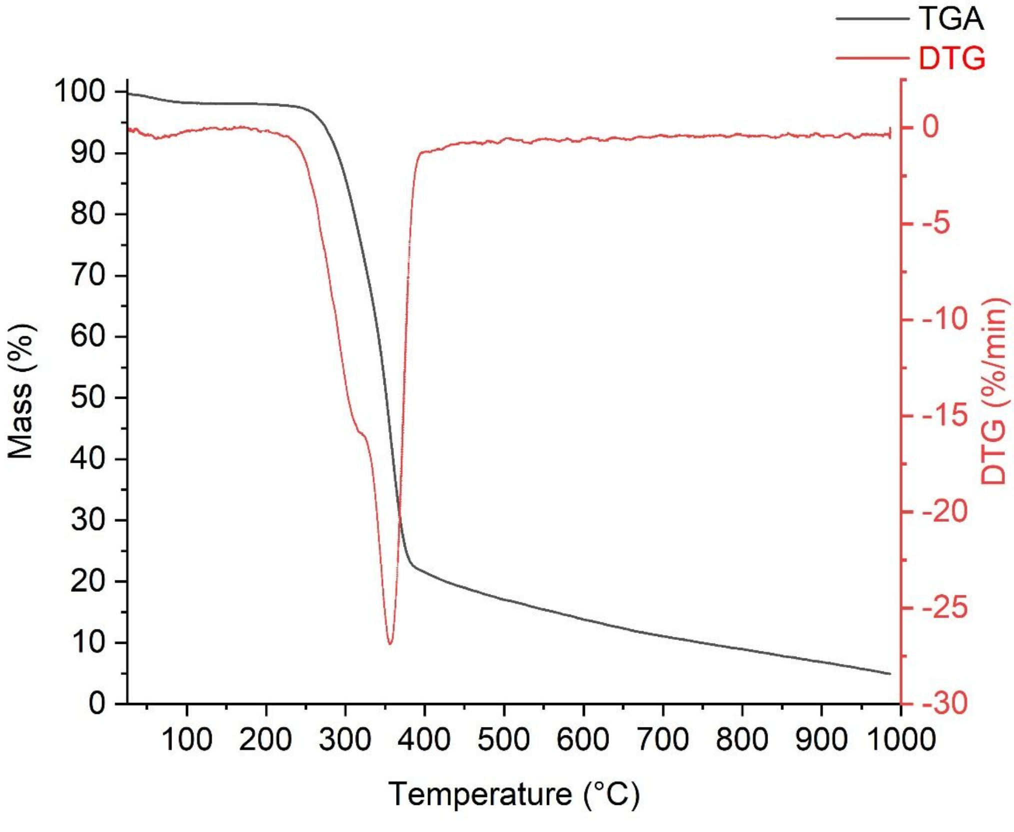

Supplement: Supplementary file 1 — ao4c02050_si_001.pdf [file ao4c02050_si_001.pdf]
